# Modeling the Cumulative Effects of Social Exposures on Health: Moving beyond Disease-Specific Models

**DOI:** 10.3390/ijerph10041186

**Published:** 2013-03-25

**Authors:** Heather L. White, Patricia O’Campo, Rahim Moineddin, Flora I. Matheson

**Affiliations:** 1 Institute of Health Policy, Management and Evaluation, University of Toronto, Toronto, ON M5T 3M6, Canada; E-Mail: heatherlynn.white@mail.utoronto.ca; 2 Institute for Clinical Evaluative Sciences, Toronto, ON M4N 3M5, Canada; E-Mails: rahim.moineddin@utoronto.ca (R.M.); mathesonf@smh.ca (F.I.M.); 3 Centre for Research on Inner City Health, The Keenan Research Centre in the Li Ka Shing Knowledge Institute, St. Michael’s Hospital, Toronto, ON M5C 1N8, Canada; 4 Dalla Lana School of Public Health, University of Toronto, Toronto, ON M5T 3M7, Canada; 5 Department of Population, Reproductive and Family Health, Johns Hopkins Bloomberg School of Public Health, Baltimore, MD 21205, USA; 6 Department of Family and Community Medicine, University of Toronto, Toronto, ON M5G 1V7, Canada

**Keywords:** social determinants, socioeconomic position, multinomial regression, multilevel modeling, health inequities

## Abstract

The traditional explanatory models used in epidemiology are “disease specific”, identifying risk factors for specific health conditions. Yet social exposures lead to a generalized, cumulative health impact which may not be specific to one illness. Disease-specific models may therefore misestimate social factors’ effects on health. Using data from the Canadian Community Health Survey and Canada 2001 Census we construct and compare “disease-specific” and “generalized health impact” (GHI) models to gauge the negative health effects of one social exposure: socioeconomic position (SEP). We use logistic and multinomial multilevel modeling with neighbourhood-level material deprivation, individual-level education and household income to compare and contrast the two approaches. In disease-specific models, the social determinants under study were each associated with the health conditions of interest. However, larger effect sizes were apparent when outcomes were modeled as compound health problems (0, 1, 2, or 3+ conditions) using the GHI approach. To more accurately estimate social exposures’ impacts on population health, researchers should consider a GHI framework.

## 1. Introduction

Over the past several decades, social epidemiologists have built an empirical base for an expanding set of etiologic factors that contribute to poor health emanating from the social, political and economic environments [1,2,3,4,5,6,7]. Explanatory models which once treated social considerations like socioeconomic position and ethnic diversity as little more than nuisance variables have rapidly expanded, allowing researchers to quantify cumulative health effects given a range of baseline conditions [8,9,10,11,12,13]. Yet integrating these new cumulative risk models within the traditionally dominant “disease-specific” models of epidemiology and medicine pose a unique set of challenges.

Disease-specific models are useful and appropriate for understanding determinants of a particular condition of interest and for targeting effective interventions for that condition. However, the exclusive focus in traditional epidemiologic inquiry on single outcome analyses is overly narrow. Such models under-estimate the true impact of adverse exposures like poverty or ethnic density because they do not consider other outcomes they may affect. For example, when examining the relationship between poverty and the presence of ischemic heart disease, those who do not have the disease may still have other conditions stemming from poverty which lie on the causal pathway, such as hypertension, diabetes, low physical activity levels, poor diet and high levels of stress. In disease-specific models, these individuals are classified into the “well” category; as a result, the cumulative risk of poverty is underestimated. Social epidemiologists need explanatory models that will allow them to accurately assess combined health effects resulting from the diverse and dynamic array of social exposures people encounter in their normal daily lives. Accurate estimates of effect magnitude are critical to informing population-level actions and policies designed to prevent or counteract adverse social exposures. In addition, comparing the magnitude of negative impact across social exposures can facilitate decision making by targeting actions and funding toward exposures with larger contributions to untoward outcomes [14]. 

Several theoretical models have been developed to offset shortfalls of the disease-specific framework. The Cumulative Risk Assessment (CRA) model has emerged from the Environmental Sciences literature as a population-based framework which can be used to estimate combined risks to health developing from an existing environmental state [12]. Several different approaches using both stressor-based (bottom-up) and effects-based (top-down) assessment have been proposed for the health sciences, however appropriate methodology and analytic evaluation of cumulative risk is still being developed [15,16]. Likewise, the Allostatic Load (AL) model has been extended from the biomedical to the social sciences for evaluating the effects of chronic stress on physical and mental health. While conceptually it extends the literature on stress and health, definitions of allostatic load as a measure remain controversial and models are still primarily applied to the study of single health outcomes [13].

In this paper we develop a “generalized health impact” (GHI) model to assess the consequences of a social exposure, socioeconomic position, on a composite health outcome encompassing 6 cumulative conditions. The GHI models extend the work of Aneshensel on social consequence models [17] to examine how social exposures impact overall health as opposed to being etiological to a particular condition. While these models share the same exposures of interest, conceptually and operationally they differ substantially [17]. The disease-specific models identify etiologic factors for a specific disorder in order to prevent or treat it. The generalized health impact model, which addresses the overall cumulative effect of social exposures, seeks to explore the “consequences of various social arrangements on people’s lives [17,18,19].” Explanations emanating from the social sciences suggest that social exposures, such as excess stress, poverty, precarious housing, or social disorganization, lead to a spectrum of host responses that may not be specific to a particular health condition [17,19,20,21,22]. In GHI models, both sociological and epidemiological theory inform the range of health conditions that might be simultaneously examined as consequence to the social exposures of interest—similar to research on social and health inequities that document the impact of social inequities on population well being [23,24]. These could include behavioral consequences such as substance dependency and/or physical consequences (heart disease, diabetes) so long as each is independently established within the literature as being a consequence of the social exposure(s) of interest.

In this paper we apply the GHI framework to model the cumulative effects of socioeconomic position (SEP) on compound health problems using a national sample of Canadians participating in three waves of the Canadian Community Health Survey [25]. We then compare estimates generated from the GHI model to those generated from traditional disease-specific models to illustrate how GHI might be considered as an alternative analytic approach in the study of social health inequities [26,27]. 

## 2. Methods

Data were obtained from two sources collected by Statistics Canada. Individual-level data came from the Canadian Community Health Survey (CCHS), a nationally representative cross-sectional survey which collects information on health determinants, outcomes and health service utilization among non-institutionalized Canadian adults [25]. Data from three waves of the CCHS were combined (2001, 2003, and 2005 surveys) to create a large dataset of 400,055 respondents representative of the general Canadian population, excluding individuals residing on aboriginal reserves, military bases, or in institutional settings [28,29,30]. The subject matter and sample populations are similar across waves and their combination allows for more efficient estimates in multilevel models by increasing the sample size captured within individual neighborhoods. Survey responses were then linked to the 2001 Canada Census by census tract (CT) using the Postal Code Conversion File Plus (PCCF4F+) [31] to define the structural characteristics of sampled urban neighbourhoods. CTs are small, relatively stable geographic units of urban populations between 2,500 and 8,000 residents, constructed similarly with respect to underlying economic status and social conditions. For practical purposes, this analysis is restricted to urban neighbourhoods as census tract characteristics are not collected for rural areas of Canada. Given our interest in assessing the social impact of SEP on health, we sought to minimize potential confounding in the occurrence of outcome events due to age and restricted the sample to respondents aged 60 and under. The combined sample thus included respondents between 18–60 years, residing in one of Canada’s 25 Urban Metropolitan Areas at the time of data collection (n = 110,031) [32]. 

To compare estimates of risk generated from disease-specific and generalized health impact models, we selected six *a priori* health conditions reflecting both physical and mental illness and addictions that have consistently shown positive associations with high levels of deprivation, low socio-economic position and chronic stress [3,6,17,19,33,34,35,36,37,38,39].

(1) *Cardiovascular disease* was defined as an affirmative answer to the question of whether respondents disclosed a current medical diagnosis or history of heart disease, hypertension or stroke.
(2) *Diabetes* was classified when individuals reported having a current medical diagnosis of diabetes.
(3) *Obese* respondents were defined as having a body mass index equal to or greater than 30, calculated from self-reported height and weight.
(4) *Heavy smokers* were defined according to guidelines developed by the United States Department of Health and Human Services as current smokers who reported consuming 10 or more cigarettes per day [40],
(5) *Heavy drinkers* were defined according to the Centers for Disease Control and Prevention as male respondents who reported consuming more than 21 drinks in the week prior to being surveyed and female respondents who consumed more than 14 drinks [41,42].
(6) *Mood disorders* were considered to be present in individuals who reported having current medical diagnosis of depression, anxiety or other mood disorders including mania, bipolar or dysthemia.

In disease-specific models, each of these health conditions were treated as separate outcome measures. However, as the focus of this analysis is on understanding how socioeconomic position impacts the cumulative burden of disease, the number of health conditions described above were summed for each respondent to provide a nominal indicator of their *compound health conditions* categorized as: 0, 1, 2 or 3+ conditions [43,44,45,46]. 

### Exposure

Three measures of socioeconomic status are used to account for low SEP exposure. *Neighbourhood deprivation*, a contextual variable, is a sub-index of the Canadian Marginalization Index [47] and reflects the level of material deprivation present within a neighbourhood [47,48]. Briefly, the index was constructed using a weighted factorial approach described previously and is comprised of the following six census measures expressed as proportions: population aged 20+ without high school graduation; lone parent families; individuals receiving government transfer payments; the population aged 15+ who are unemployed; the population living below the low income cut off (adjusted for community size, family size and inflation); and homes needing major repairs. The deprivation index is continuous in nature ranging from a score of −2 which represents a neighbourhood with low deprivation to a score of +6 which represents a neighbourhood with high deprivation. At the individual-level, *annual household income* (not adjusted for family size), was measured in the following categories: $0 to $34,999; $35,000 to $74,999 and $75,000 or greater. Respondents were also grouped according to their highest level of *education attainment*: post-secondary graduation, some post-secondary education; high school graduation and some high-school education. Control variables included *age*, *gender*, *marital status*, *ethnicity*, *survey cycle*, and *self-perceived stress*. Previous work has suggested that high levels of stress may be a significant etiologic factor for several of the adverse health outcomes under investigation [6,49,50,51,52,53]. Respondents who reported that life was either “quite a bit stressful” or “extremely stressful” were categorized and included as having excess or *high perceived stress*. 

Records with missing values were removed (n = 27,862) leaving a final sample of 82,151 respondents living in 3,644 urban neighbourhoods. There were no differences between removed cases and those retained for analysis by age, gender or SEP (multiple imputations were performed previously for missing values on income using the CCHS data. The estimated parameters based on multiple imputation—SAS Proc MI with five imputed series—were similar to estimates obtained from the model with missing cases). Ethics approval was obtained from the Research Ethics Boards of St. Michael’s Hospital and Statistics Canada data publication guidelines were followed throughout the analysis.

## 3. Statistical Analysis

Descriptive statistics were produced to describe the sample population, stratified by the number of compounded health problems and frequencies were weighted using the sampling weights provided by Statistics Canada. To allow for the hierarchical clustering of individuals living within neighbourhoods, multilevel models were used to address the research questions [54]. Multilevel logistic regression was used to examine the likelihood of reporting each health condition individually (no *vs*. yes) using a traditional disease-specific framework. A two-level multinomial regression model was then used to model the cumulative burden of disease (0 *vs*. 1, 2 and 3+ conditions) based on the formulation of Hartzel *et al.* [55]. Finally, we calculated population attributable fractions (PAF) to compare the etiologic fractions attributed to low SEP between the disease-specific and generalized health impact models. PAFs were based on the weighted proportion of cases at each exposure level and adjusted odds ratios using the following formula [56]:

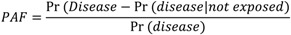


Multilevel logistic and multinomial regressions were performed using SAS NLMIXED and SAS version 9.1 (SAS Institute Inc., Cary, NC, USA) was used for data manipulation and analysis. 

## 4. Results

Table 1 provides descriptive statistics on the sample characteristics by the number of compound health conditions. The majority of respondents reported having none of the six conditions (58.2%), 29.3% reported one, 9.7% two, and 2.8% three or more conditions. Compound health problems varied by age: among those reporting no conditions, a substantially greater proportion fell into the youngest age group (18–44 years old; 71%), compared to 40.5% of those with three or more conditions who were 18–44 years old. Men and women appeared to report similar rates of individual health problems; however the number of compound health problems in the sample did vary by gender, with more women reporting multiple health conditions (data not shown). Non-Caucasian individuals were less likely to report multiple health conditions relative to Caucasians. The condition with the highest prevalence was heavy smoking (20.2% of men, 16.4% of women), followed by obesity (15.7% of men; 14.0% of women), mood disorders (9.2% of men; 16.1% of women), and CVD (11.5% of men; 10.1% of women). Higher levels of educational attainment were seen among those reporting no health conditions compared to those reporting 3+ conditions. A similar gradient was seen for household income, where lower incomes were overrepresented among those reporting 3+ conditions compared to those reporting no conditions. 

Table 2 displays results from the series of multilevel regression models. Models 1–6 show the results of logistic regression using a disease-specific approach for each health problem modeled individually, while model 7 shows the multilevel multinomial regression model based on GHI, for the reported number of compound health problems. The multilevel multinomial regression compared those with 1, 2, or 3+ conditions to those who had none of the six conditions. 

Examining the fully-adjusted disease-specific models, the odds ratios for a one-unit increase in neighborhood deprivation ranged from 1.01 (CI = 0.99−1.04) for mood disorder to 1.28 (CI = 1.25−1.31) for heavy smoking. For the GHI model, as we move from 1 to 3+ conditions, we see a trend of increasing odds ratios for neighborhood deprivation ranging from 1.13 (CI = 1.10−1.15) for one health problem to 1.33 (CI = 1.27−1.39) for 3+ compound health problems. When we estimated the unadjusted odds of reporting health problems by neighbourhood deprivation, neighbourhood deprivation was associated with a 19% increased risk of having one problem; a 33% increased risk of having two problems; and a 57% increased risk of having 3+ problems (data not shown). Upon adjustment for individual risk factors there were notable reductions in the odds ratios for neighbourhood deprivation, however the full models remained significant, suggesting a small independent risk of poor cumulative health resulting from increasing levels of neighborhood deprivation. 

In the disease-specific models, the odds ratios for individual educational attainment suggested a gradient effect, with the smallest odds ratios seen for respondents with some post-secondary education compared to those holding a university degree, and the greatest odds observed among those with less than high school compared to those with a university degree. We observed sharper gradients in the GHI model compared to those seen in the disease-specific models. For example, among individuals reporting 3+ conditions, those with post-secondary education had an odds ratio of 1.27 (CI = 1.10−1.47), and those without a high school diploma had an odds ratio of 3.27 (CI = 2.94−3.64) [57]. A similar gradient effect was seen for household income. For respondents reporting an annual household income of <$35,000 relative to those with incomes >$75,000, the risk of reporting a health condition in disease-specific models was increased for all conditions with the exception of heavy drinking (OR = 0.61; CI = 0.54−0.69). In the GHI model, the odds ratios for an income of <$35,000 for having two conditions was 1.92 (CI = 1.79−2.06), and for having three or more health conditions the odds ratio was 2.94 (CI = 2.60−3.32) compared with respondents in the highest income bracket.

High self-perceived stress was associated with an increased risk across all health outcomes and was particularly influential for mood disorders (OR = 2.68; CI = 2.56−2.80). Self-perceived stress was also associated with increasing risk for compound health problems in the GHI model. Relative to respondents reporting low to no perceived stress, those reporting high stress were over twice as likely to report having 3+ chronic conditions (OR = 2.55, CI = 2.35−2.76).

**Table 1:** Descriptive statistics for respondents aged 18–60 by number of compound health problems. Canada 2000–2005 (n = 82,151).

	Frequency Distribution (%) **				
*Variable*	*All Adults*	*0 Condition*	*1 Condition*	*2 Conditions*	*≥3 Conditions*
**Total Respondents**	82,151 (100)	45,507 (58.2)	24,913 (29.3)	8,880 (9.7)	2,851 (2.8)
Age Group					
18–44 *	53,504 (65.7)	32,380 (71.0)	15,421 (61.8)	4,606 (52.4)	1,097 (40.5)
45–60	28,647 (34.3)	13,127 (29.0)	9,492 (38.2)	4,274 (47.6)	1,754 (59.5)
Gender					
Male *	40,244 (52.1)	21,738 (50.7)	12,554 (54.2)	4,538 (54.4)	1,414 (53.6)
Female	41,907 (47.9)	23,769 (49.3)	12,359 (45.8)	4,342 (45.6)	1,437 (46.4)
Marital Status					
Married/Common law	46,012 (64.8)	26,915 (64.4)	13,508 (63.7)	4,419 (60.1)	1,270 (56.4)
Single/Divorced/Widowed/Separated	36,039 (35.2)	18,592 (35.6)	11,405 (36.3)	4,461 (39.9)	1,581 (43.6)
Ethnicity					
Caucasian *	69,609 (79.2)	36,890 (74.8)	22,001 (84.0)	8,108 (88.4)	2,610 (90.0)
Visible Minority	12,542 (20.8)	8,617 (25.2)	2,912 (16.0)	772 (11.6)	241 (10.0)
Educational Attainment					
Postsecondary Education *	51,309 (62.2)	30,942 (67.1)	14,436 (57.8)	4,569 (50.5)	1,362 (48.0)
Some Postsecondary	7,565 (9.3)	4,162 (9.2)	2,280 (9.2)	887 (10.4)	236 (7.8)
High School Graduation	14,522(18.1)	7,304 (16.6)	4,854 (20.0)	1,795 (20.4)	569 (20.5)
Less than High School	8,755(10.4)	3,099 (7.1)	3,343 (13.0)	1,629 (18.7)	684 (23.7)
Annual Household Income (in $10,000)					
Income ≥ $75,000 *	29,233 (42.3)	18,105 (45.4)	8,057 (39.5)	2,486 (36.1)	585 (27.1)
Income $35,000–$74,999	32,548 (38.4)	17,955 (37.7)	10,138 (39.5)	3,406 (38.9)	1,049 (40.8)
Income < $35,000	20,370 (19.3)	9,447 (16.9)	6,718 (21.0)	2,988 (25.0)	1,217 (32.1)
High Self-Perceived Stress					
No *	58,033 (70.7)	33,933 (74.5)	17,089 (68.3)	5,404 (59.3)	1,607 (55.9)
Yes	24,118 (29.3)	11,574 (25.5)	7,824 (31.7)	3,476 (40.7)	1,244 (44.1)

** Unweighted frequencies with weighted proportions are shown; * Indicates the reference category.

**Table 2:** Odds ratios of reporting single or compound health problems by neighborhood deprivation and socio-economic position. Canada 2000–2005 (n = 82,151).

	Multilevel Logistic Regression for Single Health Conditions *						Model 7: Multilevel Multinomial Regression for Number of Health Problems*		
Variable	Model 1: CVD	Model 2: Diabetes	Model 3: Obesity	Model 4: Heavy Drinking	Model 5: Heaving Smoking	Model 6: Mood Disorder	Number of Conditions		
							1	2	3+
*Fixed Effects*									
Neighborhood Material Deprivation	1.07 (1.04, 1.10)	1.14 (1.09, 1.20)	1.09 (1.06, 1.12)	1.09 (1.04, 1.14)	1.28 (1.25, 1.31)	1.01 (0.99, 1.04)	1.13 (1.10, 1.15)	1.20 (1.16, 1.23)	1.33 (1.27, 1.39)
Educational Attainment									
Some Post-Secondary	0.94 (0.86, 1.03)	1.04 (0.90, 1.23)	1.07 (1.00, 1.15)	1.42 (1.26, 1.60)	1.38 (1.30, 1.48)	1.18 (1.10, 1.27)	1.15 (1.09, 1.22)	1.43 (1.31, 1.55)	1.27 (1.10, 1.47)
High School Graduation	1.37 (1.32, 1.43)	1.12 (1.00, 1.25)	1.15 (1.09, 1.21)	1.43 (1.30, 1.58)	1.83 (1.74, 1.92)	1.10 (1.04, 1.15)	1.37 (1.32, 1.43)	1.62 (1.52, 1.72)	1.68 (1.52, 1.87)
Less than High School	1.46 (1.36, 1.56)	1.50 (1.34, 1.68)	1.35 (1.27, 1.44)	1.67 (1.49, 1.88)	2.85 (2.69, 3.01)	1.35 (1.26, 1.44)	1.99 (1.88, 2.10)	2.77 (2.58, 2.98)	3.27 (2.94, 3.64)
Annual Household Income (in $10,000)									
Income $35,000–$74,999	1.14 (1.08, 1.21)	1.37 (1.23, 1.52)	1.17 (1.11, 1.22)	0.73 (0.67, 0.80)	1.42 (1.35, 1.49)	1.24 (1.18, 1.32)	1.22 (1.17, 1.26)	1.30 (1.22, 1.38)	1.63 (1.47, 1.82)
Income < $35,000	1.44 (1.34, 1.55)	1.76 (1.56, 2.00)	1.28 (1.20, 1.36)	0.61 (0.54, 0.69)	1.87 (1.76, 1.98)	1.90 (1.78, 2.03)	1.45 (1.38, 1.53)	1.92 (1.79, 2.06)	2.94 (2.60, 3.32)
High Self-Perceived Stress	1.30 (1.24, 1.37)	1.20 (1.10, 2.34)	1.16 (1.11, 1.21)	1.05 (0.97, 1.14)	1.43 (1.37, 1.48)	2.68 (2.56, 2.80)	1.41 (1.36, 1.46)	2.04 (1.94, 2.14)	2.55 (2.35, 2.76)
*Random Effects*									
Level-Two Variance ^+^	0.04 (0.01)	0.04 (0.04)	0.09 (0.01)	0.09 (0.03)	0.07 (0.01)	0.09 (0.01)			0.05 (0.01)
Model Deviance (-2LL)	50,209	20,199	67,469	24,071	71,223	58,013			163,054

CI, Confidence Interval; * Models are adjusted for survey cycle, gender, age, marital status, and visible minority; ^+^ Model variance and standard errors are reported for level two.

**Table 3:** Population attributable fraction for education and income. Canada 2000–2005 (n = 82,151).

	Compound Health Problems			Single Health Conditions					
*Variable*	* 1*	* 2*	* 3+*	*CVD*	*Diabetes*	*Obesity*	*Heavy Drinking*	*Heavy Smoking*	*Mood Disorder*
Educational Attainment									
Some Postsecondary	0.03	0.03	0.02	0.01	0.01	0.01	0.06	0.06	0.03
High School Graduation	0.05	0.08	0.08	0.02	0.01	0.01	0.04	0.04	0.01
Less than High School	0.07	0.12	0.17	0.18	0.18	0.15	0.21	0.30	0.15
Annual Household Income									
Income $35,000–$74,999	0.07	0.09	0.14	0.05	0.11	0.06	0.10	0.12	0.07
Income < $35,000	0.08	0.16	0.28	0.10	0.12	0.07	0.15	0.11	0.12

To compare effect estimates from disease-specific models to those from GHI models, we calculated Population-Attributable Fractions for two of the individual-level socioeconomic exposures (Table 3). In the GHI model, PAFs for low education (high school or less than high school) and having 2 and 3+ conditions were 0.19 and 0.23, respectively. PAFs for the disease-specific conditions for low education ranged from 0.16 for obesity and mood disorders, 0.19 for diabetes and CVD, to 0.24 for heavy drinking and 0.33 for heavy smoking. Meanwhile, PAFs for income were substantially higher in the GHI model compared to the disease-specific models reflecting the higher etiologic fraction attributed to socioeconomic position for the composite outcomes compared to disease specific models. PAFs for an income <$35,000 and having 2 and 3+ conditions were 0.16 and 0.28, respectively, while the PAFs for disease-specific models for income <$35,000 was 0.07 for obesity, 0.10 for CVD, 0.11 for heavy smoking, 0.12 for diabetes and mood disorders, and 0.15 for heavy drinking. 

## 5. Discussion

Generalized health impact models are distinct from disease-specific models as they require a theoretically informed construction of the composite outcome. In this study, we were primarily interested in physical and mental health conditions that are established consequences of poverty and low income. Thus, we extended the approach used by Aneshensel [19] to develop the GHI model which included a range of health conditions that are the consequence of the social arrangements and stressors of interest to us, thus including both physical and mental illness and addictions [3,6,9,33,34,35,36,37,38,39,58,59]. This composite outcome has the advantage of reducing misclassification by ensuring that fewer individuals in the “no problem” category are afflicted with additional unmeasured conditions arising from the social exposures of interest. The GHI approach using a composite outcome is not the same as modeling a more generalized health variable, such as self-rated health. When we modeled self-rated health as an outcome using a traditional disease-specific approach, we found that for each unit increase in neighborhood deprivation, respondents had a 11% increased risk of reporting their health as fair or poor after adjusting for similar risk factors (OR = 1.11; CI = 1.08−1.14; [60]). This estimate while similar to the odds ratios seen here among individuals reporting one health condition, is still substantially lower than the odds seen among those reporting multiple conditions (2 and 3+). 

As anticipated, the effect sizes demonstrated using individual disease-specific models were lower than those observed in the GHI model. In disease-specific models, a larger proportion of individuals with related conditions affected by the same exposure are classified into the “no problem” outcome category, masking the cumulative risk of low SEP. Our comparison of the GHI and disease-specific approaches was quite simple, focusing on one social exposure—socioeconomic position, measured at the individual, household and neighbourhood levels. The magnitude of effect and increasing gradients seen for low socioeconomic position were greatest when modeled using the GHI approach. A general pattern was apparent in the GHI model with odds ratios for having only one condition being similar to estimates obtained in the disease-specific models, The stronger effects obtained from the GHI models with cumulative poor health (2 and 3+ conditions), reflect in part, the common pathway between diseases from the exposure to end-states. 

While we included a measure of self-perceived stress to account for theoretical links to the allostatic load framework, this measure of stress does not directly capture specific stressors—like those arising from life events such as a recent death, or chronic stressors such as discrimination or job dissatisfaction. Including a more complex operationalization of stress into a GHI model might help determine whether different stressors have unique or common pathways to compound health problems. The adverse consequences of stress has been amply documented [49,50,51,61], and our analysis confirms previous findings that for disease-specific models, with the exception of heavy drinking, high perceived stress was associated with an increased risk for compound health outcomes. The effect sizes demonstrated by the GHI models more effectively illustrate the overall impact of high stress on adverse health. 

Limitations of this study include the use of self-reported information which is subject to under-reporting for some outcomes, particularly for mental health outcomes and substance use. There is also possible misclassification of the outcome variable when the questions on selected health problems were not asked of respondents in a particular survey year (e.g., depression or mood disorder was not asked in each cycle we included). This would lead to underestimation of stress and the socioeconomic exposures we examined, as those who were unable to be classified as having the condition were classified as being “well”. The composite score was not weighted for severity of illness so it is possible that the increasing gradient in the odds ratios across in the GHI model may be a marker of severity; this would be a useful topic to explore in future research. Limitations of the data prevented us from capturing physiological explanations of multiple conditions. In some instances, our measure of high stress could be considered as a consequence of low socioeconomic position and thus been included in our composite outcome. Although stress is often considered as an antecedent with intervening variables such as coping and support, we elected to include it as a control variable and examine the specific impact of SEP based on Pearlin’s early work on the stress process model [49,50]. Due to the nature of the data, we were only able to examine cross-sectional associations. Future work would benefit from longitudinal and life-course data sources, which let us estimate the contribution of social exposures to the development and progression of compound health problems. Finally, CTs, despite being widely used in neighborhood research, do not always align with “natural neighborhoods” and are of varying geographic sizes. We continue to seek methods for understanding the impact of neighbourhood deprivation on the health of rural populations. The calculations on population-attributable fractions also suggest that a GHI approach might capture a more accurate measure of the impact of the socioeconomic variables we included in our models. Certainly for income, the PAFs were larger in the GHI model for multiple outcomes compared to those PAFs observed for the disease-specific models. For education, the differences were not as great, and for two outcomes (heavy drinking and heavy smoking) the PAFs were larger than those seen for the composite outcomes. This might be expected, given that the education gradient for addictions appears to be stronger than that for health conditions. The PAF gradients for both education and income show that people with more conditions would benefit more if low education and income were eliminated as exposures. 

Social epidemiologists have been calling for better analytic tools for understanding and modeling the social determinants of health [11,62,63,64,65,66,67] as current models do not conceptually or methodologically account for how social processes and factors produce and impact poor health. Although there has been an increasing focus in research and evaluation on social exposures, epidemiologic inquiry and public health interventions still typically focus on single health outcomes like smoking or diabetes improvement. The GHI model provides an alternative approach for social epidemiologists interested in evaluating social exposures and their overall health impact. We suggest that social epidemiologists look to these models as a method of analyzing data on social exposures. 
